# Electropolymerized 4-Aminobenzoic Acid Based Voltammetric Sensor for the Simultaneous Determination of Food Azo Dyes

**DOI:** 10.3390/polym14245429

**Published:** 2022-12-11

**Authors:** Guzel Ziyatdinova, Maria Titova, Rustam Davletshin

**Affiliations:** 1 Analytical Chemistry Department, Kazan Federal University, Kremleyevskaya, 18, Kazan 420008, Russia; 2 Department of High Molecular and Organoelement Compounds, Kazan Federal University, Kremleyevskaya, 18, Kazan 420008, Russia

**Keywords:** electropolymerization, 4-aminobenzoic acid, modified electrodes, electrochemical sensors, tartrazine, sunset yellow, food analysis

## Abstract

Electrochemical sensors with polymeric films as a sensitive layer are of high interest in current electroanalysis. A voltammetric sensor based on multi-walled carbon nanotubes (MWCNTs) and electropolymerized 4-aminobenzoic acid (4-ABA) has been developed for the simultaneous determination of synthetic food azo dyes (sunset yellow FCF and tartrazine). Based on the voltammetric response of the dyes’ mixture, the optimal conditions of electropolymerization have been found to be 30-fold potential scanning between −0.3 and 1.5 V, at 100 mV s^−1^ in the 100 µmol L^−1^ monomer solution in phosphate buffer pH 7.0. The poly (4-ABA)-based electrode shows a 10.5-fold increase in its effective surface area and a 17.2-fold lower electron transfer resistance compared to the glassy carbon electrode (GCE). The sensor gives a sensitive and selective response to sunset yellow FCF and tartrazine, with the peak potential separation of 232 mV in phosphate buffer pH 4.8. The electrooxidation parameters of dyes have been calculated. Simultaneous quantification is possible in the dynamic ranges of 0.010–0.75 and 0.75–5.0 µmol L^−1^ for both dyes, with detection limits of 2.3 and 3.0 nmol L^−1^ for sunset yellow FCF and tartrazine, respectively. The sensor has been tested on orange-flavored drinks and validated with chromatography.

## 1. Introduction

Electrochemical sensors with polymeric coverages have been widely developed in modern electroanalytical chemistry. Various classes of organic compounds are used as monomers and allow conducting and insulating polymers to be obtained [1,2,3,4], both of which have received attention in electroanalysis. Electrochemical polymerization is one of the perspective approaches used to form polymeric coverage at the electrode surface due to its simplicity, rapidity, low consumption of reagents, the absence of cleaning and isolation steps i.e., the exclusion of the application of additional chemical reagents and toxic organic solvents. Moreover, electrolysis parameters allow us to control the properties of the polymeric coverage obtained, and in particular, its structure and thickness [4].

Aminobenzoic acids (ABAs) are one of a number of suitable monomers for oxidative electropolymerization due to the presence of an amino group within them. Similar to other amines, aminobenzoic acids form conductive polymers [5,6,7,8]. Among them, 4-Aminobenzoic acid (4-ABA) is most frequently studied. Poly(4-ABA) has been used as sensitive layer of electrochemical sensors for pesticide carbofuran [8], bisphenol A [9], melamine [10], and for the simultaneous determination of hydroquinone and catechol [11]. Nevertheless, polyABAs-based sensors have not received enough attention compared to other conducting polymers, such as polyaniline or polythiophene, despite showing a sensitive response to various classes of analytes. The development of a poly(4-ABA)-based electrochemical sensor for the quantification of food dyes is the perspective of this study.

**Figure 1:**
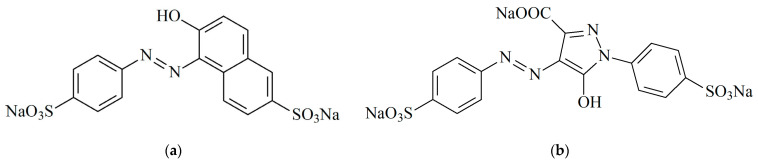
Food azo dyes structure: (**a**) Sunset yellow FCF; (**b**) Tartrazine.

Azo dyes are the most commonly used class of colorants in the food industry. The synthetic dyes sunset yellow FCF and tartrazine (Figure 1) are typical additives in beverages, jelly, and other foodstuffs [12]. Both dyes can promote various negative health effects (allergic reactions, hyperactivity in children, mutagenic and cancerogenic effects) that are dose-dependent [12,13,14,15]. Therefore, the average daily intake of sunset yellow FCF and tartrazine are set as 4 and 7.5 mg/kg_bw_, respectively [15,16], and their contents in foodstuffs and beverages must be controlled.

Sunset yellow FCF and tartrazine are electrochemically active compounds that can oxidize and reduce at the electrode surface due to the presence of hydroxy- and azo-groups, respectively. Therefore, various electrochemical approaches have been developed for the individual and simultaneous determination of these dyes [17,18,19]. Most of these approaches use chemically-modified electrodes based on nanomaterials [18]: carbon nanomaterials [20,21,22,23,24], metal [25,26] and metal oxide nanoparticles [27,28,29,30], as well as their composites and combinations [31,32,33,34,35], including graphene wrapped-phosphotungstic acid hybrid [36], and the combination of reduced graphene oxide with metal-organic frameworks of Ni with 1,3,5-benzene tricarboxylic acid [37]. An original approach for the simultaneous quantification of sunset yellow FCF and tartrazine has recently been developed using a paper-based electrode (conductive ink based on graphite powder and nail polish supported on cardboard) [38].

A limited number of polymer-modified electrodes applicable for the determination of sunset yellow FCF and tartrazine have been reported, to date (Table 1). Amino acids, particularly L-cysteine, are the most often used monomer. The oxidative electropolymerization in the potentiodynamic mode is used for the polymeric coverage formation [39,40,41,42,43,44,45,46]. Both reduction and oxidation currents can be successfully used as analytical signals. Moreover, various electrode processes have been used for the determination of sunset yellow FCF and tartrazine at the poly(L-phenylalanine)/GCE [44], which, in fact, makes their simultaneous determination impossible. The application of nanomaterials (metal and metal oxide nanoparticles or carbon nanotubes) as a platform for the polymeric coverage electrodeposition provides lower limits of detection and wider linear dynamic ranges [42,46]. Another way to improve the analytical characteristics of dyes is the application of adsorptive preconcentration at the open circuit potential [39,44]; however, this significantly increases the measurement time. The most impressive characteristics are obtained when both approaches are used, as shown on the electrode modified with polypyrrole-decorated oxidized single-walled carbon nanotubes [47]. Nevertheless, modifier synthesis is time-consuming (more than 15 h) and requires several cleaning and drying steps. A combination of nanomaterials with polymeric coverages possesses high electrical conductivity, a large surface area, and a fast electron transfer rate [48]. Therefore, the further development of a simple and fast method of direct simultaneous determination of sunset yellow FCF and tartrazine using a polymer-modified electrode is of practical interest.

The current work is focused on the creation of a voltammetric sensor based on the layer-by-layer combination of multi-walled carbon nanotubes (MWCNTs) and electropolymerized 4-ABA for the simultaneous quantification of sunset yellow FCF and tartrazine. Electropolymerization conditions that provide the best voltammetric characteristics of dyes in the simultaneous presence have been found. The effectivity of the electrode developed in electron transfer has been confirmed. The electrooxidation of sunset yellow FCF and tartrazine at the poly(4-ABA)/MWCNTs/GCE has been studied and the quantitative parameters have been calculated. The poly(4-ABA)-modified electrode acts as a sensitive and selective voltammetric sensor for azo dyes. Sensor validation was performed on orange-flavored drinks through an independent chromatographic method.

## 2. Materials and Methods

### 2.1. Reagents

Sunset yellow FCF (98% purity) from Aldrich (Steinheim, Germany), 85% tartrazine from Sigma (St. Louis, MO, USA) and 99% 4-ABA from Sigma-Aldrich (Steinheim, Germany) were used. Ascorbic acid of 99% purity (Sigma, Steinheim, Germany), 99% sorbic and 99.5% citric acids, 99% caffeine, 99% sodium benzoate, 98% niacin, and 99% inositol (Sigma-Aldrich, Steinheim, Germany) were used in the interference study. First, 10 mM standard solutions of all compounds were prepared in distilled water. The exact dilution was used for the preparation of less concentrated solution before measurements.

MWCNTs (outer diameter 40–60 nm, inner diameter 5–10 nm, and 0.5–500 μm length) from Aldrich (Steinheim, Germany) were used as an electrode surface modifier. Sonication, for 30 min in an ultrasonic bath (WiseClean WUC-A03H (DAIHAN Scientific Co., Ltd., Wonju-si, Korea), was applied for the preparation of homogeneous 0.5 mg mL^−1^ suspension of MWCNTs in 1% sodium dodecylsulfate (Panreac, Barcelona, Spain).

Other chemicals were c.p. grade and used as received. The laboratory temperature was (25 ± 2 °C).

### 2.2. Apparatus

Electrochemical measurements were conducted on the potentiostat/galvanostat Autolab PGSTAT 302N with the FRA 32M module (Eco Chemie B.V., Utrecht, The Netherlands) and NOVA 1.10.1.9 software (Eco Chemie B.V., Utrecht, The Netherlands).

A glassy electrochemical cell of 10 mL volume was used for the electrochemical measurements. A glassy carbon electrode (GCE) of 3 mm diameter (BASi^®^ Inc., West Lafayette, IN, USA) or modified electrodes were used as a working electrode and a platinum wire as an auxiliary electrode. The potentials were measured vs. an Ag/AgCl reference electrode.

The pH measurements were carried out on the “Expert-001” pH meter (Econix-Expert Ltd., Moscow, Russian Federation) with a glassy electrode.

A Merlin^TM^ high-resolution field emission scanning electron microscope (Carl Zeiss, Oberkochen, Germany) was applied for electrode surface morphology characterization and operated at a 5 kV accelerating voltage and a 300 pA emission current.

### 2.3. Procedures

#### 2.3.1. Electrode Surface Modification

The surface of the GCE was thoroughly polished on 0.05 µm alumina slurry and rinsed with acetone and distilled water. Then, 2 µL of MWCNTs suspension was drop casted.

Subsequently, 4-ABA electropolymerization was performed in potentiodynamic mode. Five scans of the supporting electrolyte (phosphate buffer pH 7.0) were recorded to achieve a stable blank curve. Then, an aliquot of 4-ABA solution was added and electropolymerization was performed. The monomer concentration, number of scans, and electrolysis parameters were optimized.

#### 2.3.2. Electrochemical Measurements

Voltammetric measurements. The supporting electrolyte (0.1 mol L^−1^ phosphate buffer of various pH) was inserted into the electrochemical cell and five scans were performed. Then, an aliquot of the sunset yellow, tartrazine, or their mixture was added. The total volume of the solution in the electrochemical cell was 5.0 mL. Cyclic or differential pulse voltammograms were registered from 0.0 to 1.2 V. The potential scan rate of 100 mV s^−1^ was set in cyclic voltammetry. The pulse parameters were varied. A baseline correction, in the NOVA 1.10.1.9 software (Eco Chemie B.V., Utrecht, The Netherlands), was applied for the calculation of the oxidation currents in the differential pulse mode.

Chronoamperometry was performed in the presence of 1.0 mM hexacyanoferrate(II) ions in 0.1 M KCl at 0.45 V for 75 s.

Electrochemical impedance spectroscopy (EIS) was carried out in the presence of a 1.0 mM mixture of hexacyanoferrate(II)/(III) ions in 0.1 M KCl, in the frequency range of 10 kHz–0.04 Hz with an applied sine potential amplitude of 5 mV, at a polarization potential of 0.21 V. The potential was calculated as a half-sum of the redox peaks of hexacyanoferrate(II)/(III) ions. The impedance spectra fitting was performed using the Randles equivalent circuit in the NOVA 1.10.1.9 software.

#### 2.3.3. Real Samples Analysis

Commercially--available, orange-flavored drinks were used as real samples. Before analysis, they were degassed by sonication for 15 min and filtered through a 0.45 µm pore size nylon membrane filter. Then, voltammetric measurements were carried out in phosphate buffer pH 4.8. Next, 50 µL of the beverage was added to the electrochemical cell and the differential pulse voltammograms were recorded within the potential window of 0.30–1.20 V, with a scan rate of 20 mV s^−1^ at the modulation amplitude of 100 mV and the modulation time of 25 ms.

#### 2.3.4. Statistical Treatment

Five replicates (three replicates for chromatography and real samples analysis) were conducted. The confidence level of 0.95 was used for the statistical treatment of the data obtained. All results were expressed as the average value ± coverage interval. The random error was evaluated using the relative standard deviation. Validation of the developed and independent methods was performed using *F*- and *t*-tests.

The detection limits were calculated as 3SD_a_/*b*, where SD_a_ was the standard deviation of the calibration graph intercept and *b* was the calibration graph slope.

Regression analysis was performed using the OriginPro 8.1 software (OriginLab, Northampton, MA, USA).

## 3. Results and Discussion

### 3.1. Voltammetric Behavior of Azo Dyes at Bare GCE and MWCNTs/GCE

**Figure 2:**
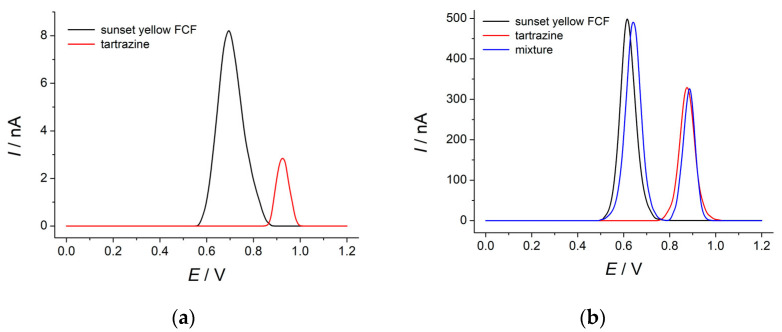
Baseline-corrected differential pulse voltammograms of 10 µmol L^−1^ sunset yellow FCF and tartrazine in phosphate buffer pH 7.0: (**a**) at the bare GCE; (**b**) at the MWCNTs/GCE. Modulation amplitude is 50 mV, modulation time is 50 ms, potential scan rate is 20 mV s^−1^.

The voltammetric behavior of sunset yellow and tartrazine in phosphate buffer pH 7.0 has been studied. Both dyes are electrochemically active at the bare GCE, at 0.695 and 0.920 V for sunset yellow FCF and tartrazine, respectively (Figure 2a). The oxidation currents are too low (approximately 8.3 and 2.8 nA for sunset yellow FCF and tartrazine, respectively at 10 µmol L^−1^), indicating the low sensitivity of the response. Furthermore, the current measurement error is very high in this case.

To overcome this limitation, electrode surface modification is needed. MWCNTs/GCE has been fabricated to solve this problem. As one can see from Figure 2b, the oxidation potentials of the dyes are shifted to lower values; by 81 mV for the sunset yellow FCF and by 44 mV for tartrazine, compared to the GCE. This is caused by the electrocatalytic effect of MWCNTs [49,50]. The oxidation currents of sunset yellow FCF and tartrazine on the modified electrode are increased by 60- and 118-fold, respectively, compared to GCE (Figure 2b). This is explained by the higher working surface area of the MWCNTs/GCE, as has been confirmed in further investigations (Section 3.3.2).

The peak potential separation of 262 mV is achieved, which makes the simultaneous detection of azo dyes (Figure 2b) possible. A slight anodic shift of the oxidation potentials for the dyes in the mixture does not affect the resolution of the signals. Nevertheless, the shape of the voltammograms suggests a partial overlapping of the signals for the higher concentration of dyes in mixture.

**Figure 3:**
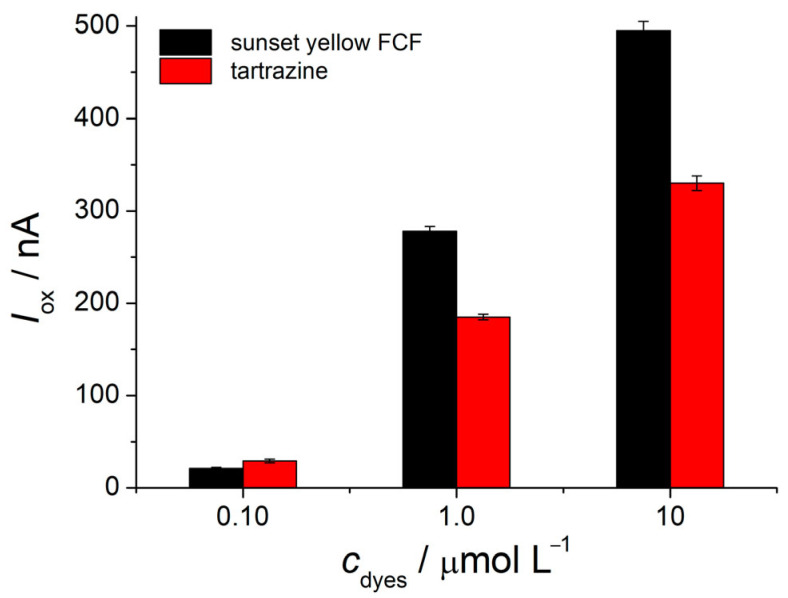
Oxidation currents of sunset yellow FCF and tartrazine mixtures of various concentrations at MWCNTs/GCE in phosphate buffer pH 7.0.

The influence of the dyes’ concentration on their voltammetric characteristics has been studied (Figure 3). A statistically significant decrease in the oxidation currents (23.3fold for the sunset yellow FCF and 11.4-fold for tartrazine) has been observed for two orders of the magnitude change in the dyes’ concentration (from 10 to 0.10 µmol L^−1^). Furthermore, the ratio of the sunset yellow FCF and tartrazine oxidation currents is also changed, while the oxidation potentials remain constant. The data obtained clearly indicate the insufficient response of MWCNTs/GCE towards sunset yellow FCF and tartrazine.

Further electrode surface modification can be applied to provide the sensitive detection of a low concentration of the azo dyes under investigation. Electropolymerized 4-ABA has been chosen.

### 3.2. Fabrication of 4-ABA-Modified Electrode

#### 3.2.1. Electropolymerization of 4-ABA at the MWCNTs/GCE

**Figure 4:**
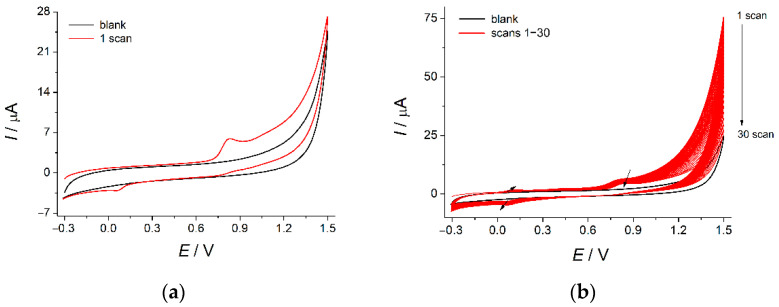
Cyclic voltammograms of 100 µmol L^−1^ 4-ABA at the MWCNTs/GCE in phosphate buffer pH 7.0: (**a**) 1 scan; (**b**) 1–30 scans. The potential scan rate is 100 mV s^−1^.

4-ABA is oxidized at the MWCNTs/GCE in phosphate buffer pH 7.0 (Figure 4a) at 0.82 V. The presence of a weakly pronounced reduction step at 0.063 V confirms the irreversibility of electrooxidation. In the following scans, the oxidation peak potential is insignificantly shifted to more positive values, while the oxidation currents gradually decreased as the number of scans increased (Figure 4b). The reduction peak at 0.063 V is reversible, which is confirmed by the appearance of the corresponding anodic peak at 0.11 V on the second scan. The redox peak currents increase with the growth in the number of potential scans, which confirms the formation of conducting coverage and agrees well with the literature [6,7]. Furthermore, an irreversible oxidation step at 0.425 V has been registered on the anodic branch of the voltammogram, which is consistent with [8].

**Scheme 1:**
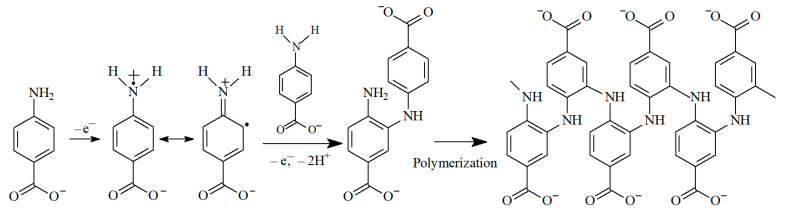
4-ABA electropolymerization.

The electrooxidation of 4-ABA at 0.82 V corresponds to the formation of cation-radical (Scheme 1 [6,7,51,52]), which undergoes further coupling reactions.

#### 3.2.2. Optimization of 4-ABA Electropolymerization Conditions

As has previously been acknowledged [4], electropolymerization conditions strongly affect the properties of final coverage and its response to the target analyte. Therefore, optimization of the 4-ABA electropolymerization conditions has been performed on the basis of the voltammetric response of the sunset yellow FCF and tartrazine mixture. The monomer concentration, the number of scans and the electrolysis parameters have all been varied.

The peak potential separation of the dyes is almost unaffected by the electropolymerization conditions and is equal to 251 mV. The oxidation currents are statistically significantly changed. Therefore, 4-ABA electropolymerization optimization has been performed by means of oxidation currents of a 0.10 µmol L^−1^ mixture of sunset yellow FCF and tartrazine.

**Figure 5:**
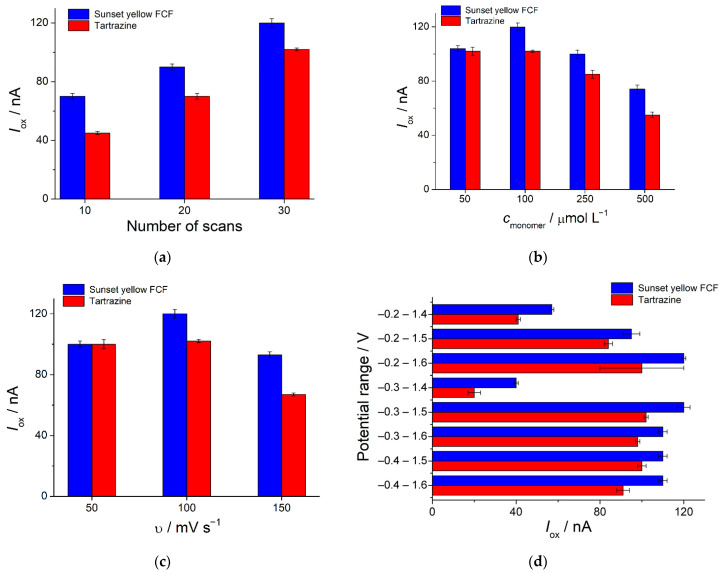
Effect of 4-ABA electropolymerization conditions on the oxidation currents of the 0.10 µmol L^−1^ mixture of sunset yellow FCF and tartrazine at the poly(4-ABA)/MWCNTs/GCE in phosphate buffer pH 7.0 in differential pulse mode: (**a**) Effect of scan number; (**b**) Effect of monomer concentration; (**c**) Effect of potential scan rate; (**d**) Effect of potential range.

Of the increase in the number of scans leads to the significant growth of both dyes’ oxidation currents (Figure 5a). Nevertheless, the use of more than 30 scans is impractical as it increases the time required for the electrode preparation and leads to significant increase in the capacitive currents, which affects the response of the azo dyes. Therefore, 30 scans were used for poly(4-ABA) formation.

The increase in the 4-ABA concentration, up to 100 µmol L^−1^ (Figure 5b), provides the growth of the sunset yellow FCF and tartrazine oxidation currents; this is caused by the thickness of the conducting coverage formed. A further increase in the monomer concentration leads to a statistically significant decrease in the dyes’ oxidation currents, most likely due to the incomplete electrooxidation of the monomer. In this sense, 30 scans were not enough in this case. This assumption has been confirmed by 250 µmol L^−1^ 4-ABA and 50 scans providing almost similar oxidation currents of the azo dyes as seen in the case of 100 µmol L^−1^ of 4-ABA and 30 scans. Nevertheless, the capacitive currents are very high, which is a negative side effect in voltammetry. Therefore, 100 µmol L^−1^ of 4-ABA and 30 scans have been used for further study.

The variation of the electrolysis parameters (Figure 5c,d) shows that the maximum oxidation currents of the dyes was achieved at the polymeric coverage, obtained at a potential scan rate of 100 mV s^−1^, in the range between −0.3 and 1.5 V and between –0.2 and 1.6 V. However, a high error for the tartrazine response has been observed in the case of the potential range between −0.2 and 1.6 V. Potential window shortening (less than 1.8 V) leads to lower oxidation currents of the sunset yellow FCF and tartrazine. This can be caused by an incomplete electropolymerization of 4-ABA. A wider potential range also leads to a decrease in the dyes’ oxidation currents, at 8–11%, which is likely caused by the overoxidation of the npolymeric coverage.

Thus, optimal conditions of 4-ABA electropolymerization that provide the best amperometric response from the sunset yellow FCF and tartrazine mixture are at a 30-fold potential scanning, between −0.3 and 1.5 V at 100 mV s^−1^ rate in the 100 µmol L^−1^ monomer solution in phosphate buffer pH 7.0.

The poly(4-ABA)-modified electrode shows electrochemical activity. There are a weakly-defined reversible redox pair of peaks at 0.111 and 0.091 V and an oxidation peak at 0.42 V on the cyclic voltammograms in phosphate buffer pH 7.0 (Figure S1). Their potentials are shifted with the pH change, indicating the participation of protons in the electrochemical reaction, which is consistent with that reported in [8].

### 3.3. Bare and Modified Electrodes Characterization

#### 3.3.1. Surface Morphology

**Figure 6:**
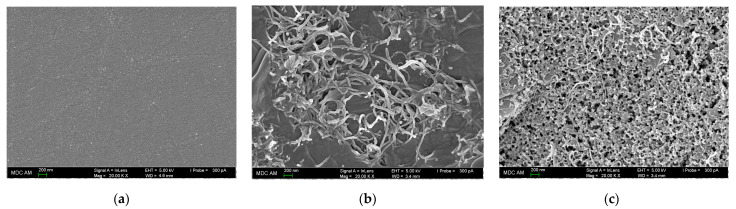
Electrode surface morphology by scanning electron microscopy: (**a**) bare GCE; (**b**) MWCNTs/GCE; (**c**) Poly(4-ABA)/MWCNTs/GCE.

The electrode’s surface morphology has been studied through scanning electron microscopy (Figure 6). The GCE shows low roughness due to its relatively smooth surface (Figure 6a). The modification of the electrode surface with MWCNTs leads to the appearance of intertwined nanotubes, of 40 to 70 nm thickness, forming a network structure. In addition, their aggregates are included in the surfactant film (Figure 6b). The poly(4-ABA) layer is presented by the irregularly shaped formations, which are evenly distributed over the electrode surface and form a porous coverage with pore sizes ranging between 60 and 110 nm (Figure 6c). This structure leads to an increase in the surface roughness and area; this is confirmed by the electrochemical data obtained in further investigations (Section 3.3.2). The scanning electron microscopy data indicate the successful formation of the polymer coating and its uniform distribution over the electrode surface.

#### 3.3.2. Effective Surface Area and Electron Transfer Properties

**Figure 7:**
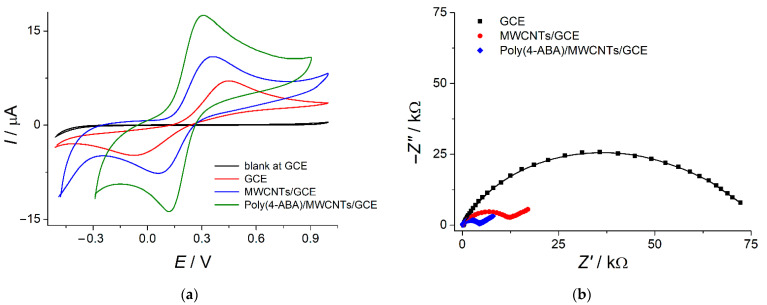
(**a**) Cyclic voltammograms of 1.0 mM hexacyanoferrate(II) ions in 0.1 M KCl at the bare GCE, MWCNTs/GCE and poly(4-ABA)/MWCNTs/GCE. The potential scan rate is 100 mV s^−1^; (**b**) Nyquist plot (experimental (points) and fitted (lines)) for bare GCE, MWCNTs/GCE and poly(4-ABA)/MWCNTs/GCE in the presence of 1.0 mmol L^−1^ hexacyanoferrate(II)/(III) ions in 0.1 mol L^−1^ KCl. *E* = 0.21 V; frequency range =10 kHz–0.04 Hz; amplitude = 5 mV.

A cyclic voltammetry of 1.0 mmol L^−1^ hexacyanoferrate(II) ions in 0.1 mol L^−1^ KCl was used for the estimation of the electrodes’ effective surface area (Figure 7a). The electrooxidation of the hexacyanoferrate(II) ions at the bare GCE proceeded, irreversibly, as the peak potential separation and cathodic to anodic currents ratio indicate. Therefore, chronoamperometry has been applied for the evaluation of the GCE effective surface area (Figure S2). Using the Cottrell equation and the slopes of the plots *I* vs. *t*^−1/2^, an 8.9 ± 0.3 mm^2^ surface has been calculated for GCE.

Electrode surface modification provided a reversible oxidation of the redox marker (Figure 7a), which confirms an increase in the electron transfer rate. The effective surface area of the MWCNTs/GCE and poly(4-ABA)/MWCNTs/GCE, of 75 ± 3 and 93.7 ± 0.9 mm^2^, was calculated using the Randles–Ševčík equation. A 10.5- and 1.25-fold increase in the effective surface area of the polymer-modified electrode vs. bare GCE and MWCNTs/GCE agree with the scanning electron microscopy data and explain the increase in the sunset yellow FCF and tartrazine oxidation currents.

EIS was used for the evaluation of the electron transfer properties. The impedance spectra are presented as Nyquist plots (Figure 7b). The significant decrease in the semicircle diameter in the high frequency range indicates an increase in the electron transfer rate at the modified electrodes. The quantitative impedance parameters (Table 2) were obtained using spectra fitting with Randles’ equivalent circuits (*R*_s_(*R*_et_*Q*) for the bare GCE and *R*_s_(*Q*[*R*_et_*W*]) of the modified electrodes, where *R*_s_ is the electrolyte resistance, *R*_et_ is the electron transfer resistance, *Q* is the constant phase element, and *W* is the Warburg impedance [53]).

A 6- and 17.2-fold decrease in the electron transfer resistance was found for the modified electrodes, compared to the bare GCE. The poly(4-ABA) layer provides another 2.9-fold decrease in the *R*_et_ value vs. MWCNTs/GCE, confirming the effectivity of the polymeric coverage in the electron transfer. The increase in the constant phase element is most likely caused by the conductivity and porous structure of the polymeric coverage. The Warburg impedance is also increased, indicating a higher electron transfer rate.

The successful formation of poly(4-ABA) at the electrode surface and its effectivity in electron transfer were confirmed using scanning electron microscopy and electrochemical methods.

### 3.4. Voltammetric Response of Dyes Mixture at the Poly(4-ABA)-Modified Electrode

**Figure 8:**
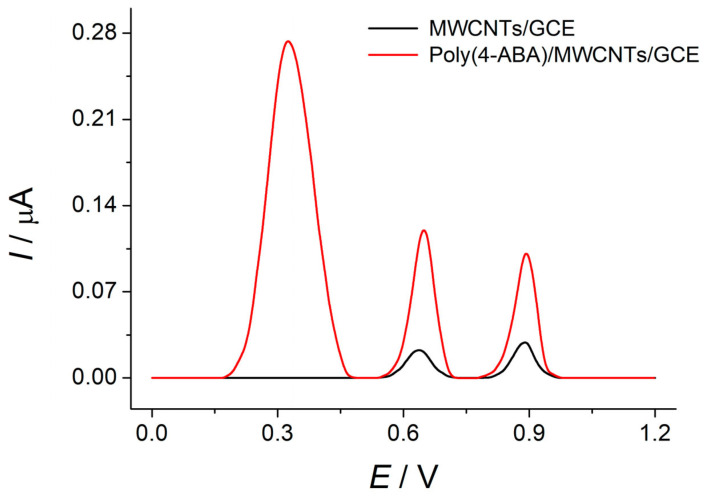
Baseline-corrected differential pulse volammograms of 0.10 µmol L^−1^ mixture of sunset yellow FCF and tartrazine at the MWCNTs/GCE and poly(4-ABA)/MWCNTs/GCE in phosphate buffer pH 7.0. Modulation amplitude is 50 mV, modulation time is 50 ms, potential scan rate is 20 mV s^−1^.

The voltammetric characteristics of the 0.10 µmol L^−1^ mixture of sunset yellow FCF and tartrazine at the poly(4-ABA)-modified electrode have been compared to those at the MWCNTs/GCE. There is a well-defined oxidation peak at 0.32 V on the differential pulse voltammograms of the supporting electrolyte (Figure 8), corresponding to polymeric coverage. Sunset yellow FCF and tartrazine are oxidized at the more positive potentials (0.65 and 0.90 V, respectively) and their oxidation peaks do not overlap with the signal of the polymeric coverage.

The oxidation potentials of the dyes at the poly(4-ABA)/MWCNTs/GCE are almost the same as at the MWCNTs/GCE, while the oxidation currents are statistically significantly increased (5.2-and 3.5-fold for sunset yellow FCF and tartrazine, respectively); this is caused by the growth of the surface area of the polymer-modified electrode.

The data obtained indicate the effectivity of the combination of poly(4-ABA) and MWCNTs as a GCE surface modifier for the simultaneous detection of food azo dyes.

### 3.5. Electrooxidation of Sunset Yellow FCF and Tartrazine at the Polymer-Modified Electrode

**Figure 9:**
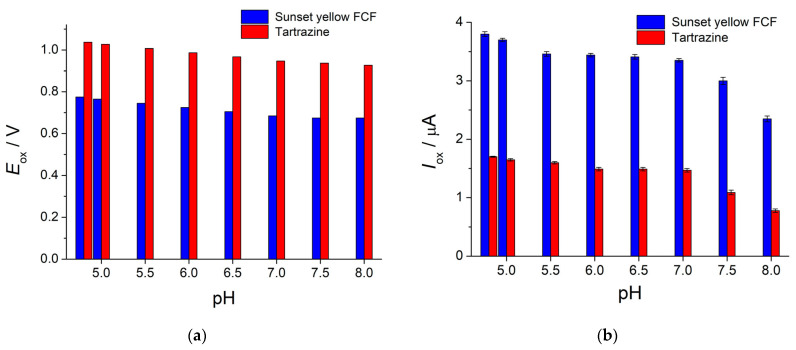
Effect of phosphate buffer pH on the voltammetric characteristics of 100 µmol L^−1^ of sunset yellow FCF and tartrazine at the poly(4-ABA)/MWCNTs/GCE: (**a**) the changes of oxidation potentials; (**b**) the changes of oxidation currents.

The electrooxidation of the azo dyes at the poly(4-ABA)/MWCNTs/GCE has been studied using cyclic voltammetry in a phosphate buffer. The effect of the pH in the range of 4.8–8.0 on the voltammetric characteristics of sunset yellow FCF and tartrazine has been investigated (Figure 9a,b). The oxidation of both dyes proceeds irreversibly in the whole pH range. The oxidation potentials are changed to lower values as the pH increased to 7.0 (Figure 9a), indicating the participation of protons in the electrode reaction; pH-independent oxidation is observed in the basic medium, which agrees well with the partial deprotonation of the hydroxyl groups in their structure [54,55,56].

The oxidation currents of both dyes are also decreased with the increase in pH (Figure 9b). The difference in the oxidation currents at a pH of 5.5–7.0 is statistically insignificant for each dye. A sharp decrease in the oxidation currents has been observed in the basic medium, which is caused by the chemical oxidation of the dyes by air oxygen. The highest signals of both dyes were obtained at pH 4.8, which has been used in further investigations. Under these conditions, sunset yellow FCF and tartrazine exist as di- and tri-anions, respectively [55].

**Figure 10:**
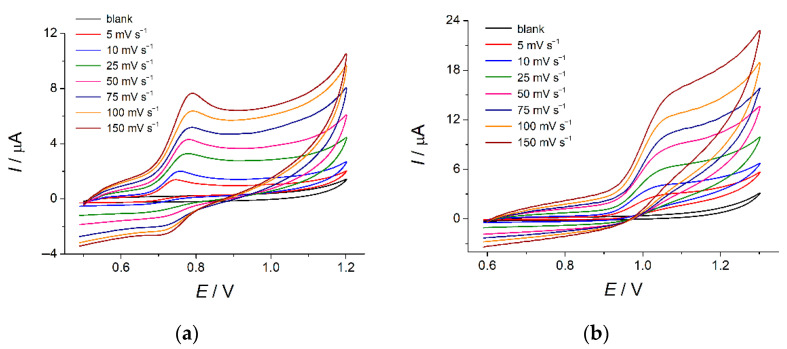
Cyclic voltammograms of azo dyes at the poly(4-ABA)/MWCNTs/GCE in phosphate buffer pH 4.8 at various potential scan rates: (**a**) 100 µmol L^−1^ of sunset yellow FCF; (**b**) 500 µmol L^−1^ of tartrazine.

A potential scan rate effect in the range of 5–150 mV s^−1^ on the dyes’ voltammetric characteristics has been studied (Figure 10).

The oxidation currents of sunset yellow FCF and tartrazine increased linearly as a function of the square root of potential scan rate (Table 3), indicating a diffusion-driven process for both dyes. This is also confirmed by the slopes of the Napierian logarithmic dependence of the oxidation currents vs. the potential scan rate (Table 3), which are 0.43 and 0.45 for sunset yellow FCF and tartrazine, respectively, and close to the theoretical value of 0.5 [57]. Such behavior of dyes has been reported at other modified electrodes [58,59].

**Scheme 2:**
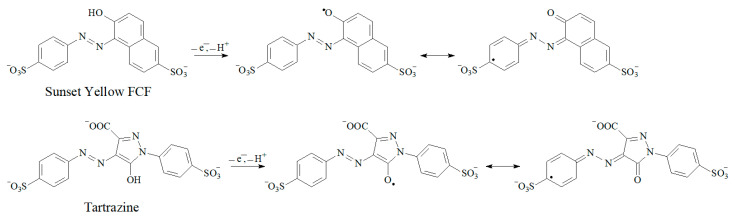
Electrooxidation of sunset yellow FCF and tartrazine.

A weak-defined step on the cathodic branch of the cyclic voltammograms of sunset yellow has been observed at the high scan rates. Tartrazine does not give a cathodic step on the voltammograms. Furthermore, the oxidation potentials of both dyes were linearly shifted in the anodic direction as the potential scan rate increased (Table 3). This behavior indicates the irreversibility of the electrode reaction and is confirmed by the anodic transfer coefficients, of 0.60 and 0.49 for sunset yellow FCF and tartrazine, respectively, which were calculated from the slopes of the Tafel plots [57]. The following equation is valid in this case (Equation 1) [57]:

(1) $$E_{p}=\left(\frac{RT}{2\alpha_{a}nF}\right)\ln\upsilon +\mathrm{const},$$

where *R* is the universal gas constant (8.314 J mol^−1^ K^−1^), *T* is the temperature (298 K), α_a_ is the anodic transfer coefficient, *n* is the number of electrons, *F* is the Faraday constant (96485 C mol^−1^), υ is the potential scan rate (V s^−1^). Based on the slopes of the *E* = *f*(lnυ) plots and the anodic transfer coefficients values, the number of electrons participating in the electrode reactions are 0.93 for sunset yellow FCF and 1.2 for tartrazine, i.e., one-electron electrooxidation takes place for both dyes. Similar data have been reported in [20,21,58,59,60]. The hydroxyl group in the azo dyes’ structure undergo one electron and one proton transfer, according to Scheme 2. The radicals formed can undergo further reactions.

The diffusion coefficients and the standard heterogeneous electron transfer rate constants of sunset yellow FCF and tartrazine have been calculated and presented in Table 4.

### 3.6. Simultaneous Determination of Azo Dyes Using Poly(4-ABA)-Based Sensor

The developed sensor was used for the simultaneous determination of sunset yellow FCF and tartrazine under the conditions of differential pulse voltammetry, which is characterized by higher sensitivity than linear sweep modes.

#### 3.6.1. Optimization of Pulse Parameters

The effect of modulation amplitude and time on the voltammetric response of the dyes’ mixture have been studied. The oxidation potentials of sunset yellow FCF and tartrazine are decreased as the modulation amplitude and time are increased (Figure S4a,b), while the peak potential separation (Figure S4c) is almost similar. The oxidation currents increase significantly with the increasing modulation amplitude. The maximum currents of both dyes were obtained at a modulation amplitude of 100 mV (Figure S4d,e). In contrast, the increase in the modulation time leads to a dramatic decrease in the sunset yellow FCF and tartrazine oxidation currents (Figure S4d,e). Therefore, the modulation amplitude of 100 mV and the modulation time of 25 ms were chosen for the dye’s quantification.

#### 3.6.2. Analytical Characteristics of Azo Dyes

**Figure 11:**
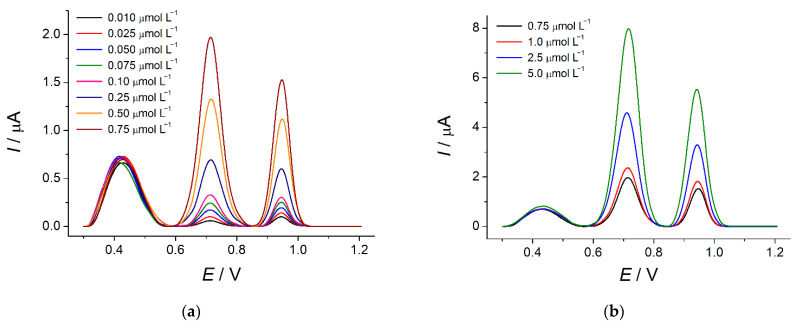
Baseline-corrected differential pulse voltammograms of sunset yellow FCF and tartrazine equimolar mixtures at the poly(4-ABA)/MWCNTs/GCE in phosphate buffer pH 4.8: (**a**) 0.010–0.75 µmol L^−1^; (**b**) 0.75–5.0 µmol L^−1^. Modulation amplitude is 100 mV, modulation time is 25 ms, potential scan rate is 20 mV s^−1^.

Sunset yellow FCF and tartrazine show well-resolved oxidation peaks at 0.713 and 0.945 V, respectively, on the differential pulse voltammograms of the dyes’ mixtures. The linear growth of the peak’s height was observed as the dyes’ concentration was increased (Figure 11 and Figure S5).

The analytical characteristics of azo dyes and corresponding calibration graphs parameters are shown in Table 5.

The linear dynamic ranges and limits of detection are among the best reported for polymer-modified electrodes to date (Table 1). Furthermore, the determination is based on the oxidation signal, which excludes the use of inert gas for the removal of oxygen affecting the reduction signals of the dyes, as was observed in [43,44]. Better analytical characteristics were obtained at the electrode modified with polypyrrole decorated oxidized single-walled carbon nanotubes [45]; however, the adsorptive preconcentration step takes 5 min, rendering the method time-consuming. In addition, this step can lead to the co-preconcentration of other components of the sample.

**Figure 12:**
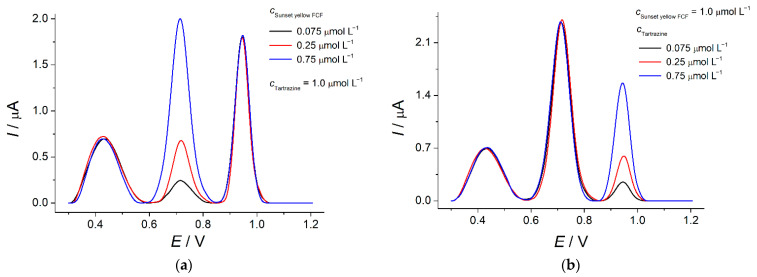
Baseline-corrected differential pulse voltammograms of sunset yellow FCF and tartrazine non-equimolar mixtures at the poly(4-ABA)/MWCNTs/GCE in phosphate buffer pH 4.8: (**a**) fixed 1.0 µmol L^−1^ concentration of tartrazine and varied concentration of sunset yellow FCF; (**b**) fixed 1.0 µmol L^−1^ concentration of sunset yellow FCF and varied concentration of tartrazine. Modulation amplitude is 100 mV, modulation time is 25 ms, potential scan rate is 20 mV s^−1^.

The investigation of non-equimolar mixtures of sunset yellow FCF and tartrazine by varying the concentration of one dye at the fixed high contents of the second one has shown the absence of the peaks’ overlap in the whole range of concentrations (Figure 12a,b). The oxidation currents increased proportionally for one dye and remained constant for the second dye. Moreover, the oxidation currents of both dyes in the non-equimolar mixtures coincided with those of the equimolar mixtures. These data confirm that the electrooxidation of sunset yellow FCF and tartrazine mixture proceeds independently. Therefore, calibration graphs for equimolar mixtures can be applied to determine sunset yellow FCF and tartrazine, both individually and in mixtures containing various ratios of dyes. Such an approach significantly simplifies the practical application of the sensor.

#### 3.6.3. Accuracy, Reproducibility, and Robustness of the Sensor Response to Azo Dyes

The accuracy of the sunset yellow FCF and tartrazine determination using a poly(4-ABA)-based sensor has been tested on their model mixtures (Table 6). Six concentration levels, covering the whole dynamic range, were used and the absence of random errors was confirmed based on the relative standard deviation values, which are less than 5.1%. The recovery values of 99–100.4% indicate the high accuracy of the developed sensor.

The oxidation currents of the azo dyes significantly decreased in the second and subsequent scans; this was most likely caused by the adsorption of the oxidation products at the surface polymer-modified electrode. Therefore, electrode surface renewal is required after each measurement, and the repeatability parameter cannot be evaluated. Thus, the relative standard deviation values (Table 6) prove the high reproducibility of the sensor’s response to azo dyes.

The robustness of the sensor was also tested. The relative standard deviation of the sunset yellow FCF and tartrazine’s oxidation currents changed at 1.5–2.0% after the replacement of the GCE; orthophosphoric and sodium hydroxide were used to obtain the phosphate buffer and the preparation of new MWCNTs suspension. Thus, the sensor developed is robust.

#### 3.6.4. Interference Study

**Figure 13:**
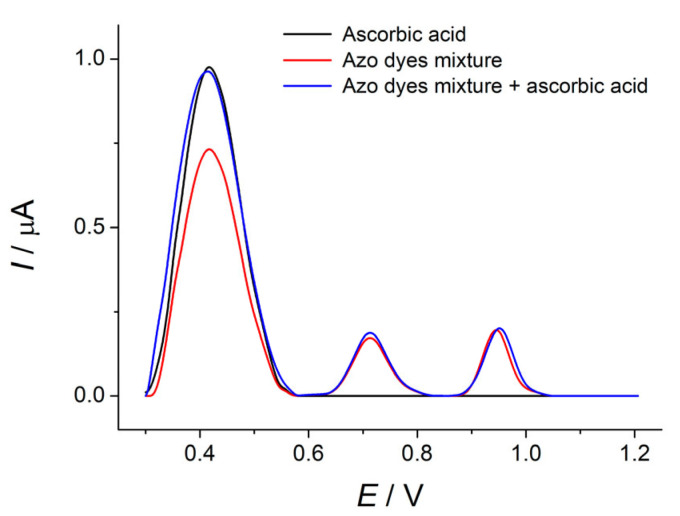
Baseline-corrected differential pulse volammograms of 0.05 µmol L^−1^ mixture of sunset yellow FCF and tartrazine in the absence and in the presence of 50 µmol L^−1^ ascorbic acid at the MWCNTs/GCE and poly(4-ABA)/MWCNTs/GCE in phosphate buffer pH 4.8. Modulation amplitude is 100 mV, modulation time is 25 ms, potential scan rate is 20 mV s^−1^.

The selectivity of the electrochemical sensor’s response to target analytes is a key point of possibility for its practical application. The effect of various potential interferences on the response of the 0.050 µmol L^−1^ mixture of sunset yellow FCF and tartrazine at the poly(4-ABA)-based sensor has been evaluated. Inorganic ions (K^+^, Mg^2+^, Ca^2+^, Al^3+^, NO_3_^−^, Cl^−^, and SO_4_^2−^), saccharides (glucose, rhamnose, and sucrose) do not oxidize in the potential range considered. Their 1000-fold excesses do not affect the oxidation potentials and currents of sunset yellow FCF and tartrazine. Other typical components in soft drinks and beverages are ascorbic, sorbic and citric acids, caffeine, sodium benzoate, niacin and inositol; these have been tested at a 0.10 mmol L^−1^ level. All of these, excluding ascorbic acid, were electrochemically inactive at the poly(4-ABA)/MWCNTs/GCE under the conditions of the experiment. The response of unset yellow FCF and tartrazine response did not change in the presence of their 100-fold excess, indicating the absence of an interfering effect. As for the ascorbic acid, it is electroactive at the electrode developed, as the significant increase in the blank oxidation peak at 0.43 V was observed after the addition of the ascorbic acid (Figure 13). The peak shape remains the same; thus, no overlap between the oxidation peaks of sunset yellow FCF and tartrazine was observed up to a 100-fold excess of ascorbic acid in the mixture (Figure 13). Thus, the poly(4-ABA)-based sensor shows high selectivity towards sunset yellow FCF and tartrazine and can be used in the analysis of real samples.

### 3.7. Practical Application of the Sensor

The developed poly(4-ABA)-based sensor was successfully applied for the analysis of orange-flavored drinks. All samples contained both dyes, whose oxidation peaks have been clearly defined in the corresponding voltammograms (Figure S6). The identity of the oxidation peaks to the dyes has been confirmed by the oxidation potential values and by the standard addition method (Figure S6). The proportional increase in the oxidation currents was observed following the addition of the dyes to the sample in the electrochemical cell. The recovery values (Table 7) indicate the absence of matrix effects.

The analysis data of the real samples are summarized in Table 8. The developed sensor has been validated with the chromatographic method [61]. One sample *t*-test was used for the characterization of the systematic errors and *F*-test for the evaluation of the equality of the two variances. The data obtained confirm the absence of systematic errors in the determination of the sunset yellow FCF and tartrazine, as well as confirming that the variances of two populations are homogeneous at α = 0.05 and that there are no significant differences in the precision of voltammetry and chromatography.

## 4. Conclusions

A novel voltammetric sensor based on the electropolymerized 4-ABA has been developed for the sensitive and selective simultaneous quantification of sunset yellow FCF and tartrazine. The conditions of the potentiodynamic electropolymerization were optimized on the basis of the voltammetric response of the target azo dyes. The effectivity of poly(4-ABA) as a sensitive layer in electrochemical sensors has been confirmed by voltammetry and electrochemical impedance spectroscopy. 

The investigation carried out expands the application of poly(4-ABA)-based sensors in organic analysis, particularly in terms of the determination of food dyes. The analytical capabilities of the sensor in food quality control have been demonstrated. Further development can be focused on the fabrication of screen-printed electrodes modified with MWCNTs as a platform for the immobilization of polymeric coverage.

## Figures and Tables

**Table 1:** Polymer-based voltammetric sensors for sunset yellow and tartrazine determination.

Sensor	Dye	Method	Redox Process	*t*_acc_ (s)	Detection Limit (µmol L^−1^)	Linear Dynamic Range (µmol L^−1^)	Ref
Poly(L-cysteine)/GCE ^1^	Sunset yellow FCF	DPV ^2^	Oxidation	300	0.0040	0.0080–0.70	[39]
Poly(L-cysteine)/PGE ^3^				—	0.125	1.0–1000	[40]
Polyglycine/CPE ^4^	Tartrazine	CV ^5^	Oxidation	—	0.283	1–27 and 35–87	[41]
Poly (*p*-aminobenzenesulfonic acid)/ZnO nanoparticles-CPE		DPV		—	0.080	0.0349–1.246 and 1.246–5.44	[42]
Electropolymerized hemin/GCE	Sunset yellow FCF	SWV^6^	Reduction	—	0.384	0.5–10	[43]
	Tartrazine			—	0.360	0.5–10	
Poly(L-phenylalanine)/GCE	Sunset yellow FCF	DPV	Reduction	60	0.040	0.40–14	[44]
	Tartrazine		Oxidation	80	0.020	0.40–20 and 20–165	
Poly(L-methionine)/Reduced graphene oxide/SPCE ^7^	Sunset yellow FCF	DPV	Oxidation	300	0.048	1–10 and 10–50	[45]
	Tartrazine			420	0.041	1–10 and 10–85	
Poly(L-cysteine)/Ag nanoparticles/GCE	Sunset yellow FCF	DPV	Oxidation	—	0.075	0.50–10 and 10–300	[46]
	Tartrazine			—	0.25	0.75–75 and 75–750	
Polypyrrole-SWCNTs-COOH ^8^/GCE	Sunset yellow FCF	SWV	Oxidation	300	0.00070	0.0050–1.0	[47]
	Tartrazine			300	0.0010	0.0070–1.0	

^1^ Glassy carbon electrode. ^2^ Differential pulse voltammetry. ^3^ Pencil graphite electrode. ^4^ Carbon paste electrode. ^5^ Cyclic voltammetry. ^6^ Square-wave voltammetry. ^7^ Screen printed carbon electrode. ^8^ Carboxylated single-walled carbon nanotubes.

**Table 2:** Fitted parameters of the electrochemical impedance (*n* = 5; *p* = 0.95).

Electrode	*R*_s_ (Ω)	*R*_et_ (kΩ)	*Q* (µΩ^−1^)	*n*	*W* (µΩ^−1^)	χ^2^
GCE	245 ± 5	72 ± 3	3.7 ± 0.2	0.789	–	0.028
MWCNTs/GCE	199 ± 3	12.1 ± 0.9	3.6 ± 0.1	0.775	230 ± 5	0.015
Poly(4-ABA)/MWCNTs/GCE	113 ± 2	4.18 ± 0.05	389 ± 4	0.913	246 ± 3	0.020

**Table 3:** Effect of the potential scan rate on the voltammetric characteristics of sunset yellow FCF and tartrazine at the poly(4-ABA)/MWCNTs/GCE in phosphate buffer pH 4.8.

Dye	Linear Regression Parameters		
	*a* ± SD	*b* ± SD	*R* ^2^
	***I* (µA) = *a* + *b*υ^1/2^ (mV s^−1^)**		
Sunset yellow FCF	0.28 ± 0.05	0.301 ± 0.007	0.9970
Tartrazine	0.18 ± 0.04	0.332 ± 0.005	0.9989
	**ln*I* (µA) = *a* + *b*lnυ (V s^−1^)**		
Sunset yellow FCF	2.17 ± 0.04	0.43 ± 0.01	0.9967
Tartrazine	2.29 ± 0.02	0.45 ± 0.01	0.9990
	***E* (V) = *a* + *b*lnυ (V s^−1^)**		
Sunset yellow FCF	0.842 ± 0.003	0.023 ± 0.001	0.9960
Tartrazine	1.109 ± 0.005	0.022 ± 0.002	0.9834

**Table 4:** Diffusion coefficients (*D*) and the standard heterogeneous electron transfer rate constants (*k*^0^) of the sunset yellow FCF and tartrazine at the poly(4-ABA)/MWCNTs/GCE in phosphate buffer pH 4.8.

Parameter	Equation	Sunset Yellow FCF	Tartrazine
*D* (cm^2^ s^−1^)	Iox=π12χ(bt)nFAcD12(αanαFRT)12υ12	(1.06 ± 0.07) × 10^−6^	(1.91 ± 0.06) × 10^−7^
*k*^0^ (cm s^−1^)	k0=2.415e−0.02FRTD12(Ep−Ep/2)−12υ12	6.4 × 10^−4^	2.1 × 10^−4^

**Table 5:** Analytical characteristics and calibration graph parameters for sunset yellow FCF and tartrazine determination using a poly(4-ABA)/MWCNTs-based sensor.

Dye	Limit of Detection (µmol^−1^ L)	Linear Dynamic Range (µmol^−1^ L)	*I* = *a* + *bc*		
			*a* ± SD (µA)	(*b* ± SD) × 10^−5^ (µA mol^−1^ L)	*R* ^2^
Sunset yellow FCF	0.0023	0.010–0.75	0.039 ± 0.002	26.14 ± 0.07	0.9999
		0.75–5.0	0.99 ± 0.06	14.1 ± 0.2	0.9993
Tartrazine	0.0030	0.010–0.75	0.096 ± 0.002	20.08 ± 0.06	0.9999
		0.75–5.0	0.93 ± 0.04	9.2 ± 0.1	0.9994

**Table 6:** Simultaneous determination of sunset yellow FCF and tartrazine in model mixtures at the poly(4-ABA)/MWCNTs/GCE in phosphate buffer pH 4.8 (*n* = 5; *p* = 0.95).

Dye	Added (µg)	Found (µg)	RSD (%)	*R* (%)
Sunset yellow FCF	0.0226	0.0227 ± 0.0004	1.6	100 ± 2
	0.113	0.113 ± 0.006	5.1	100 ± 5
	0.226	0.227 ± 0.004	1.5	100 ± 2
	1.13	1.13 ± 0.02	1.5	100 ± 2
	2.26	2.27 ± 0.02	0.91	100.4 ± 0.9
	11.3	11.32 ±0.08	0.54	100.2 ± 0.7
Tartrazine	11.3	11.32 ± 0.08	2.9	100.2 ± 0.7
	0.0267	0.0263 ± 0.0009	4.9	99 ± 3
	0.134	0.134 ± 0.007	1.9	100 ± 5
	0.267	0.265 ± 0.006	2.0	99 ± 2
	1.34	1.34 ± 0.03	2.2	100 ± 2
	13.4	13.4 ± 0.1	0.64	100.0 ± 0.7

**Table 7:** Recovery of sunset yellow FCF and tartrazine in orange-flavored drinks at the poly(4-ABA)/MWCNTs/GCE in phosphate buffer pH 4.8 (*n* = 5; *p* = 0.95).

Sample	Dye	Spiked (µmol L^−1^)	Found (µmol L^−1^)	RSD (%)	*R* (%)
Sample 1	Sunset yellow FCF	0	0.144 ± 0.005	1.4	
		0.072	0.21 ± 0.01	2.9	97 ± 3
		0.144	0.289 ± 0.005	0.69	100 ± 2
	Tartrazine	0	0.37 ± 0.01	1.9	
		0.186	0.55 ± 0.01	0.92	98 ± 2
		0.37	0.74 ± 0.02	1.0	100 ± 3
Sample 2	Sunset yellow FCF	0	0.60 ± 0.01	0.92	
		0.30	0.89 ± 0.03	1.2	99 ± 3
		0.60	0.120 ± 0.004	1.3	100 ± 3
	Tartrazine	0	0.17 ± 0.01	2.9	
		0.085	0.255 ± 0.009	2.0	100 ± 3
		0.17	0.343 ± 0.006	0.73	101 ± 2

**Table 8:** Quantification of sunset yellow FCF and tartrazine in orange-flavored drinks (*p* = 0.95).

Sample	Dye	Dye Contents (mg L^−1^)				*t*-Test *	*F*-Test **
		Voltammetry	RSD (%)	Chromatography	RSD (%)		
1	Sunset yellow FCF	6.5 ± 0.2	1.3	6.7 ± 0.5	5.0	0.634	15.5
	Tartrazine	19.6 ± 0.5	1.7	20.0 ± 0.9	1.8	1.38	1.14
2	Sunset yellow FCF	26.9 ± 0.6	0.97	27 ± 1	2.1	0.575	4.56
	Tartrazine	9.2 ± 0.7	2.9	9 ± 1	8.3	0.279	7.86
3	Sunset yellow FCF	27 ± 1	1.6	28 ± 3	4.9	0.153	9.44
	Tartrazine	7.8 ± 0.7	3.4	7.9 ± 0.5	2.7	0.609	1.52

* *t*_crit_ = 2.78 at α = 0.05 and *f* = 4. ** *F*_crit_ = 19.00 at α = 0.05 and *f*_1_ = *f*_2_ = 2.

## Data Availability

The data presented in this study are available in the electronic Supplementary Materials.

## Supplementary Materials

The following supporting information can be downloaded at: https://www.mdpi.com/article/10.3390/polym14245429/s1, Figure S1: Cyclic voltammogram of poly(4-ABA)/MWCNTs/GCE in phosphate buffer pH 7.0. Potential scan rate is 100 mV s^−1^; Figure S2: (a) Chronoamperometric curves of hexacyanoferrate(II) ions in 0.1 M KCl at the bare GCE at 0.45 V; (b) Plot of *I* vs. *t* ^−½^ on the basis of chronoamperometric data; Figure S3: Cyclic voltammograms of 100 µmol L^−1^ of azo dyes at the poly(4-ABA)/MWCNTs/GCE in phosphate buffer of various pH: (**a**) sunset yellow FCF; (**b**) tartrazine. Potential scan rate is 100 mV s^−1^. Figure S4: Effect of modulation amplitude and time on the voltammetric characteristics of 1.0 µmol L^−1^ mixture of azo dyes at the poly(4-ABA)/MWCNTs/GCE in phosphate buffer pH 4.8: (**a**) changes in the oxidation potential of sunset yellow FCF; (**b**) changes in the oxidation potential of tartrazine; (**c**) changes in the peak potential separation of dyes; (**d**) changes in the oxidation currents of sunset yellow FCF; (**e**) changes in the oxidation currents of tartrazine; Figure S5: Calibration plots of azo dyes at the poly(4-ABA)/MWCNTs/GCE in phosphate buffer pH 4.8: (**a**) Sunset yellow FCF; (**b**) Tartrazine; Figure S6: Baseline-corrected differential pulse voltammograms of 50 µL of orange-flavored drinks at the poly(4-ABA)/MWCNTs/GCE in phosphate buffer pH 4.8: (**a**) Sample 1 (curve 1), sample 1 + 0.072 µmol L^−1^ of sunset yellow FCF and 0.185 µmol L^−1^ of tartrazine (curve 2), sample 1 + 0.144 µmol L^−1^ of sunset yellow FCF and 0.37 µmol L^−1^ of tartrazine (curve 3); (**b**) Sample 2 (curve 1), sample 2 + 0.30 µmol L^−1^ of sunset yellow FCF and 0.085 µmol L^−1^ of tartrazine (curve 2), sample 2 + 0.60 µmol L^−1^ of sunset yellow FCF and 0.17 µmol L^−1^ of tartrazine (curve 3). Modulation amplitude is 100 mV, modulation time is 25 ms, potential scan rate is 20 mV s^−1^.
