# Supplementary material for: Electropolymerized 4-Aminobenzoic Acid Based Voltammetric Sensor for the Simultaneous Determination of Food Azo Dyes
Source: Polymers (Basel). 2022 Dec 11;14(24):5429. doi: 10.3390/polym14245429 (PMC9783049; doi:10.3390/polym14245429)
Supplement: Supplementary file 1 [file polymers-14-05429-s001.zip › polymers-2067724-supplementary.pdf]

Supplementary Material

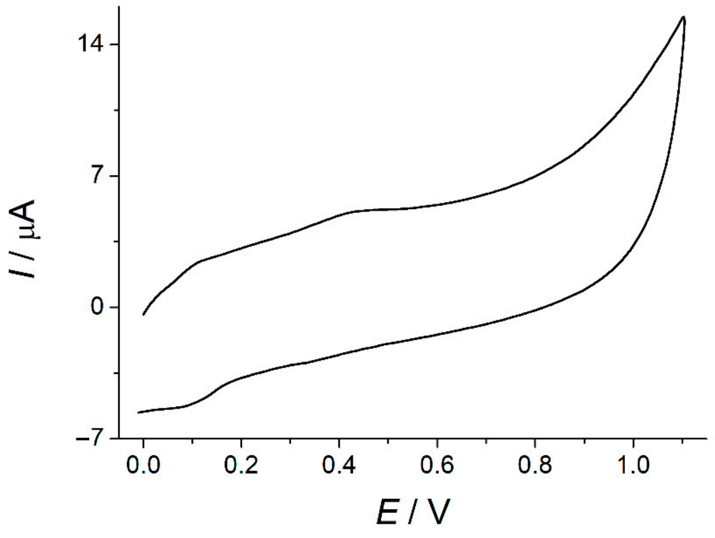

Figure S1. Cyclic voltammogram of poly(4-ABA)/MWCNTs/GCE in phosphate buffer pH 7.0. Potential scan rate is 100 mV s^{-1}.

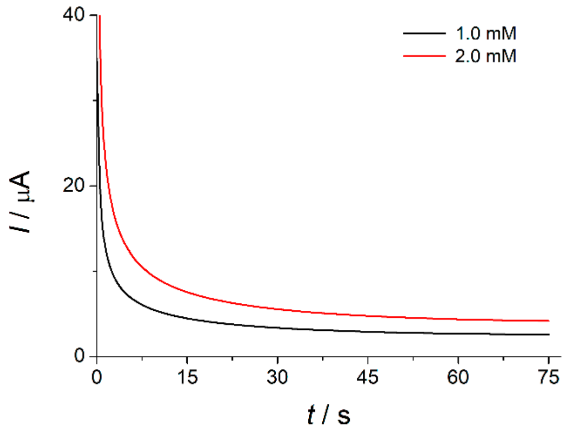

(**a**)

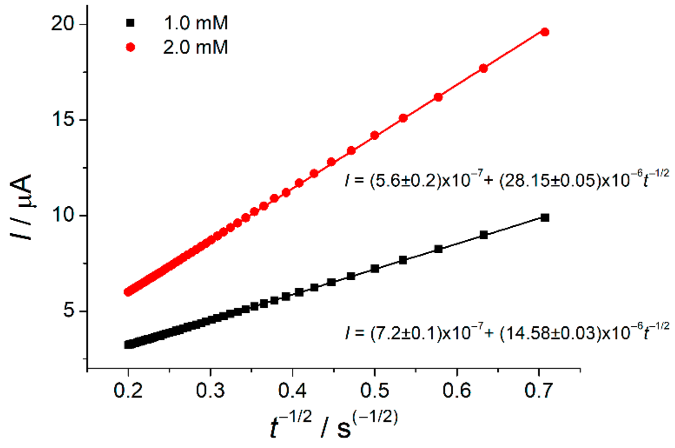

(**b**)

Figure S2. (**a**) Chronoamperometric curves of hexacyanoferrate(II) ions in 0.1 M KCl at the bare GCE at 0.45 V; (**b**) Plot of I vs. $t^{-1/2}$ on the basis of chronoamperometric data.

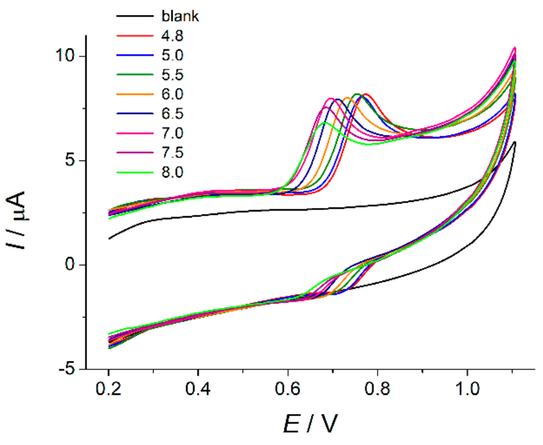

(**a**)

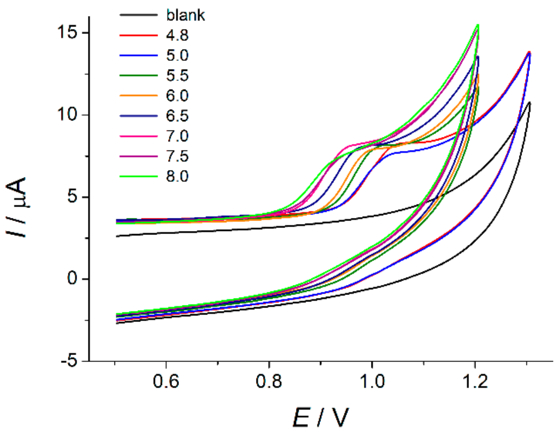

(**b**)

Figure S3. Cyclic voltammograms of 100 μmol L^{-1} of azo dyes at the poly(4-ABA)/MWCNTs/GCE in phosphate buffer of various pH: (**a**) sunset yellow FCF; (**b**) tartrazine. Potential scan rate is 100 mV s^{-1}.

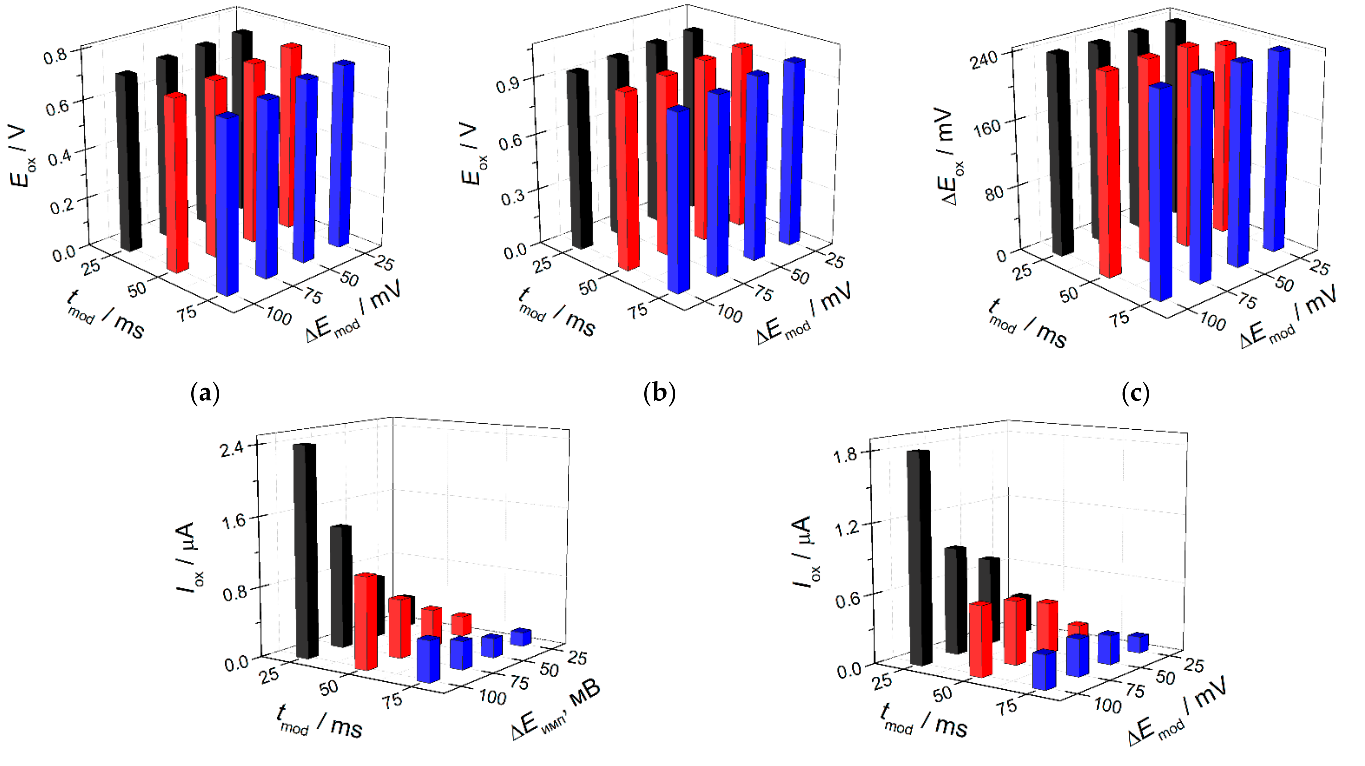

(**d**) (**e**)

Figure S4. Effect of modulation amplitude and time on the voltammetric characteristics of 1.0 μmol L^{-1} mixture of azo dyes at the poly(4-ABA)/MWCNTs/GCE in phosphate buffer pH 4.8: (**a**) changes in the oxidation potential of sunset yellow FCF; (**b**) changes in the oxidation potential of tartrazine; (**c**) changes in the peak potential separation of dyes; (**d**) changes in the oxidation currents of sunset yellow FCF; (**e**) changes in the oxidation currents of tartrazine.

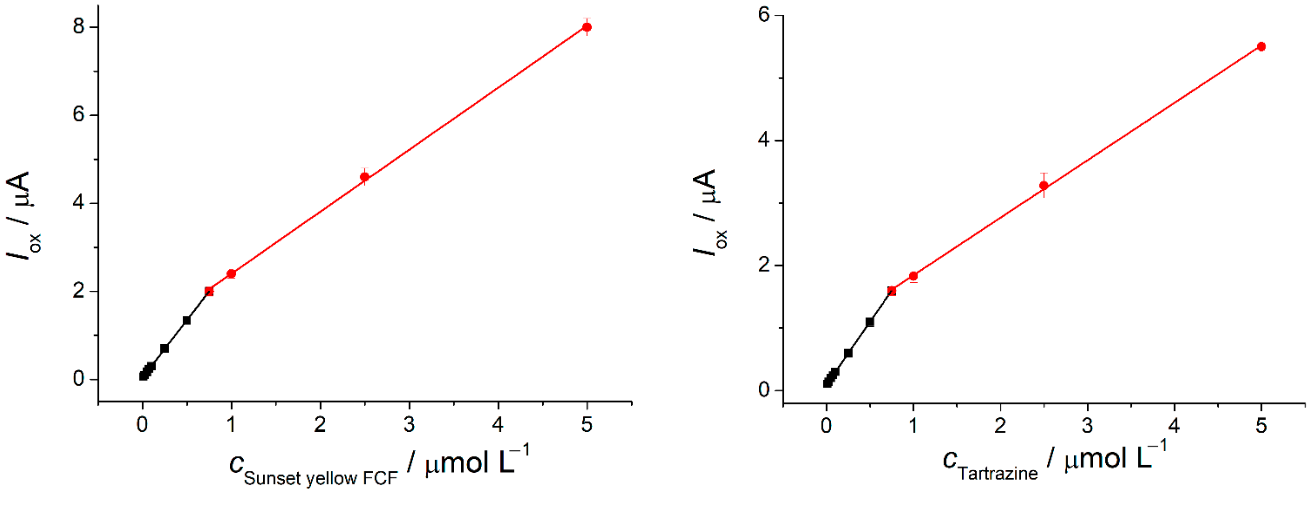

(**a**) (**b**)

Figure S5. Calibration plots of azo dyes at the poly(4-ABA)/MWCNTs/GCE in phosphate buffer pH 4.8: (**a**) Sunset yellow FCF; (**b**) Tartrazine.

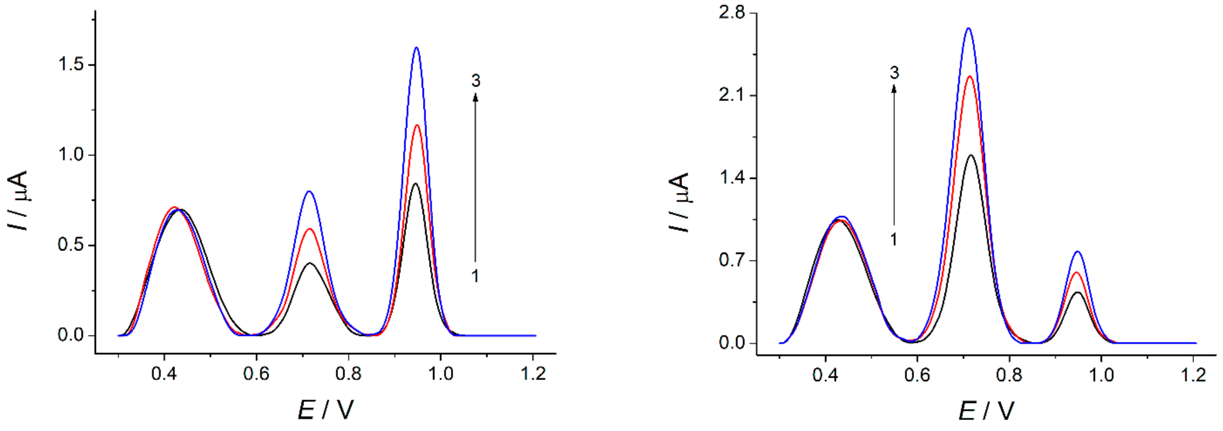

(**a**) (**b**)

Figure S6. Baseline-corrected differential pulse voltammograms of 50 μL of orange-flavoured drinks at the poly(4-ABA)/MWCNTs/GCE in phosphate buffer pH 4.8: (**a**) Sample 1 (curve 1), sample 1 + 0.072 μmol L^{-1} of sunset yellow FCF and 0.185 μmol L^{-1} of tartrazine (curve 2), sample 1 + 0.144 μmol L^{-1} of sunset yellow FCF and 0.37 μmol L^{-1} of tartrazine (curve 3); (**b**) Sample 2 (curve 1), sample 2 + 0.30 μmol L^{-1} of sunset yellow FCF and 0.085 μmol L^{-1} of tartrazine (curve 2), sample 2 + 0.60 μmol L^{-1} of sunset yellow FCF and 0.17 μmol L^{-1} of tartrazine (curve 3). Modulation amplitude is 100 mV, modulation time is 25 ms, potential scan rate is 20 mV s^{-1}.
